# Parry's *Extra-Uterine Pregnancy*

**Published:** 1885-06

**Authors:** 


*Extra-uterine Pregnancy.* By JOHN S. PARRY. Pp. 276. London: H. K. LEWIS. 1876.

THIS admirable work, by a Philadelphia physician, published nine years ago, is probably the best monograph on Extra-uterine Pregnancy in existence. The labour bestowed upon it must have been immense. The work is based upon a collection of no fewer than five hundred cases, and these by no means exhaust the literature which the writer has waded through and sifted. Now that the question of operative treatment in extra-uterine pregnancy is coming prominently to the front, this work of Dr. Parry's must come to have a new and increased importance. The surgeon, anxious to be familiar with everything that is known concerning a condition for which he may, at any moment, be called upon to operate, will find exactly what he wants in this book. For the recent advances in treatment by Laparotomy, he must have recourse to scattered records. A new edition would be gratefully received.

